# *Xenopsylla* spp. (Siphonaptera: Pulicidae) in murid rodents from the Canary Islands: An update

**DOI:** 10.1051/parasite/2012194423

**Published:** 2012-11-15

**Authors:** S. Sánchez, M.S. Gómez

**Affiliations:** Department of Microbiology and Parasitology, Faculty of Pharmacy, University of Barcelona Avda. Joan XXIII 08028 Barcelona Spain

**Keywords:** *Xenopsylla* spp., *Mus musculus*, *Rattus rattus*, distribution, Canary Islands, *Xenopsylla spp.*, *Mus musculus*, *Rattus rattus*, distribution, Îles Canaries

## Abstract

The geographical and host distributions of *Xenopsylla* fleas parasitizing murid rodents on the Canary Islands have been reported. Three *Xenopsylla* species, *X. cheopis*, *X. brasiliensis* and *X. guancha*, have been detected on two rodents species, *Mus musculus* and *Rattus rattus*. *X. guancha* has been the most prevalent species detected, specifically on *M. musculus*, the most abundant rodent, but it has been detected only on three eastern islands, where the species is endemic. *X. cheopis* has been shown to be the most widely distributed species throughout the archipelago and the species most frequently found on *R. rattus*. *X. brasiliensis* has been shown to be the least prevalent *Xenopsylla* species, with the lowest geographical distribution on the Canary Islands and focused only on *R. rattus*. The detection of both *X. cheopis* and *X. brasiliensis* on the island of Lanzarote, and of *X. guancha* on the island of Fuerteventura and the islet of La Graciosa represents the first report of these species on those particular Canary Islands.

## Abstract (fr)

On rapporte la distribution géographique et les espèces de rongeurs muridés parasités par *Xenopsylla* dans les Îles Canaries. Trois espèces de *Xenopsylla*, *X. cheopis*, *X. brasiliensis* et *X. guancha*, ont été détectées sur deux rongeurs, *Mus musculus* et *Rattus rattus*. *X. guancha* a été la puce la plus abondante bien qu’elle ne parasite que *M. musculus;* toutefois, cet hôte a été de loin le plus étudié dans l’ensemble de l’archipel et *X. guancha* n’a été collectée que sur les trois îles orientales. *X. cheopis* a été l’espèce la plus répandue dans l’archipel et plus fréquemment détectée sur *R*. *rattus*. *X. brasiliensis* a été la puce la moins abondante, la moins répandue et ne parasitant que *R. rattus*. C’est la première fois que l’on détecte *X. cheopis* et *X. brasiliensis* dans l’île de Lanzarote, de même que *X. guancha* à Fuerteventura et sur l’îlot de La Graciosa.

The Canary Islands form the southernmost Palearctic archipelago, located at latitude and longitude 27º 29º N and 13º 18º W, respectively. On the archipelago, field-rats as well as house-rats are found along with house mice. *Rattus rattus* (Linnaeus, 1758) and *Mus musculus* (Linnaeus, 1758) are the most abundant sylvatic rodents, and precisely murid species on these islands.

Four *Xenopsylla* species have been reported on the Canary Islands, three of them, *X. brasiliensis* (Baker, 1904), *X. cheopis* (Rothschild, 1903) and *X. guancha*Beaucournu, Alcover & Launay, 1989 on murid rodents, and *X. gratiosa* (Jordan and Rothschild, 1923) on sea-birds (Hopkins & Rothschild, 1953; Beaucournu & Launay, 1990). *Xenopsylla* species are essentially indigenous to the Ethiopian Region, parasitizing mainly rodents over their entire geographic distribution (Hopkins & Rothschild, 1953; Lewis, 1972). *X. cheopis* and *X. brasiliensis* have spread from the Ethiopian Region to other warm parts of the world by rats carried on ships. Among *Xenopsylla* species present in Europe, two of them, *X. cunicularis* (Smit, 1957) and *X. gratiosa*, are parasites of warm-blooded animals other than rodents, respectively rabbit and petrel.

Reports on the flea fauna of the Canary Islands have been, until now, scarce and rather old (Gil Collado *et al.*, 1982; Zapatero et al., 1982; Beaucournu *et al.*, 1989; Beaucournu & Launay, 1990). As part of a series of research projects conducted over a period of six years and dealing with the ectoparasitic and endoparasitic of theriological fauna of these islands, this article reports on the geographical and host distribution of *Xenopsylla* species detected on the archipelago.

## Material and Methods

**Fig. 1.**
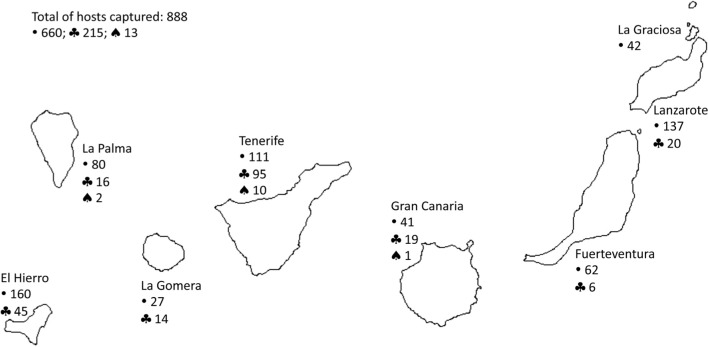
Host distribution in each Island of the Archipelago. • *Mus musculus*; ♣ *Rattus rattus*; ♠ *Rattus norvegicus*.

During the course of these projects, seven main islands and one islet have been prospected; four western (El Hierro, La Palma, La Gomera and Tenerife), and four eastern (Gran Canaria, Fuerteventura, Lanzarote and La Graciosa). In each island, different biotopes were prospected throughout a week, and, in average, 200 live-capture traps were used every night. As is displayed in Fig. 1, a total of 888 murid rodents – 660 *M. musculus*, 215 *R. rattus* and 13 *R. norvegicus* (Berkenhout, 1769) – were trapped and then they were scarified by cervical dislocation.

Flea specimens detected on rodents were kept in 70 % alcohol. Each specimen was subjected to a clearing treatment with 20 % potassium hydroxide and to a process of dehydration, using a series of ethanol rinses of ascending concentration up to 100 %. Finally, for identification purposes, the specimens were mounted in Canada balsam.

The specific and sub-specific flea determination was based on their morphological characteristics (Hopkins & Rothschild, 1953; Beaucournu *et al.*, 1989; Beaucournu & Launay, 1990).

## Results and Discussion

None of the 13 *R. norvegicus* captured was parasitized by *Xenopsylla* fleas. The three murids *Xenopsylla* species, *X. cheopis*, *X. brasiliensis* and *X. guancha*, already reported on the Canary Islands were also detected in this study. A total of 98 *Xenopsylla* spp. fleas (59 ♂, 39 ♀) were found on 61 rodents (a prevalence of 6.9 % and 1.6 of mean intensity).

43 *X. cheopis* (24 ♂, 19 ♀) were found on 22 rodents, four *X. brasiliensis* (3 ♂, 1 ♀) on three rodents and 51 *X. guancha* (32 ♂, 19 ♀) on 36 rodents. The global prevalence and mean intensity for all the rodents trapped over the entire archipelago was: 2.5 % and 1.9 for *X. cheopis*; 0.3 % and 1.3 for *X. brasiliensis*; 4.0 % and 1.4 for *X. guancha*. *Xenopsylla* fleas were absent on the rodents from La Palma and La Gomera (Table I).

**Table I.** Results about *Xenopsylla* spp. in the Canary Islands.

		El Hierro			La Palma			La Gomera			Tenerife			Gran Canaria			Fuerteventura			Lanzarote			La Graclosa			Total			
*Xenopsylla*	Hostspecies	T(♂, ♀)	P	MI	T(♂, ♀)	P	MI	T(♂, ♀)	P	MI	T(♂, ♀)	P	MI	T(♂, ♀)	P	MI	T(♂, ♀)	P	MI	T(♂, ♀)	P	MI	T(♂, ♀)	P	MI	T(♂, ♀)	P	MI
*X.cheopis*	*Musmusculus*	5 (1, 4)	2.5	1.3	–	–	–	–	–	–	1	0.9	1.0	1	2.4	1.0	2	4.8	1.0	–	–	–	–	–	–	9	1.4	1.0
	*Rattusrattus*	21 (10, 11)	13.5	3.5	–	–	–	–	–	–	–	–	–	7 (6, 1)	21.1	1.8	5 (4,1)	33.3	2.5	1 (1,0)	5.0	1.0				34 (21, 13)	6.0	2.6
	*Rattusnorvegicus*				–	–	–				–	–	–	–	–	–										–	–	–

*X.brasiliensis*	*Musmusculus*	–	–	–	–	–	–	–	–	–	–	–	–	–	–	–	–	–	–	–	–	–
	*Rattusrattus*	–	–	–	–	–	–	–	–	–	–	–	–	1 (1, 0)	5.3	1.0	–	–	–	4 (3, 1)	1.4	1.3
	*Rattusnorvegicus*				–	–	–				–	–	–	–	–	–										–	–	–

*X. guancha*	*Musmusculus Rattusrattus*	–	–	–	–	–	–	–	–	–	–	–	–	–	–	–	8 (4, 4)	6.5	1.5	38 (26, 12)	19.7	1.4	5 (2, 3)	11.9	1.0	51 (32, 19)	5.5	1.4
	*Rattusrattus*	–	–	–	–	–	–	–	–	–	–	–	–	–	–	–	–	–	–	–	–	–				–	–	–
	*Rattusnorvegicus*				–	–	–				–	–	–	–	–	–										–	–	–

T (♂, ♀) = total number of *Xenopsylla* (males and females) in each island for each rodent host; P= prevalence of *Xenopsylla* in each Island for each rodent host; MI: mean intensity.

In this study, as well as in previous studies, *R. rattus* was found to be the most parasitized host by *X. cheopis* (6.0 %, Table I) (Zapatero Ramos *et al.*, 1982; Beaucournu *et al.*, 1989). This flea species was the most widespread, present on five of the seven main islands studied: on El Hierro, Gran Canaria and Fuerteventura on both rodent hosts, on Tenerife only on *M. musculus* and on Lanzarote on *R. rattus*. *X. brasiliensis* was present only on the islands of Gran Canaria and Lanzarote and always on a *R. rattus* host, although specimens of *M. musculus* were also captured there (1.4 %, Table I). *X. guancha* was the most prevalent species on the whole archipelago (4.0 %) but was focused on *M. musculus* (5.5 %, Table I) the most abundant everywhere and, also, on the three easternmost islands of the archipelago (Fuerteventura, Lanzarote and La Graciosa).

The actual detection of *X. cheopis* on Lanzarote represents the first report of this species on the island. It should be pointed out that El Hierro was the island on which *X. cheopis* was most abundant but restricted to a biotope, which is a dry xerophytic scrub located 75 meters above sea level, where this species was parasitizing all rodents trapped (six *R. rattus* and two *M. musculus*). As in previous studies, *X. cheopis* was absent on two of the western islands, La Palma and La Gomera, and also on one of the eastern, La Graciosa islet (Zapatero Ramos *et al.*, 1982; Beaucournu *et al.*, 1989; Beaucournu & Launay, 1990). The current and previous absence of *X. cheopis* on La Palma and La Gomera is curious, because no ecological or host explanation exists. The physiographic characteristics of these islands are quite similar to those of the other western islands (El Hierro and Tenerife), where the flea species was found and its two rodent hosts were trapped.

In this study, *X. brasiliensis* was the least prevalent *Xenopsylla* species and also the least widespread flea on the archipelago. The presence of this species had been reported on three western (La Palma, La Gomera and Tenerife) and on one eastern Canary Island (Gran Canaria) and also only on *R. rattus* (Nájera, 1942; Beaucournu *et al.*, 1989). The present results reveal the presence of *X. brasiliensis* on Lanzarote for the first time, and, because the rat populations were less abundant than the mouse populations on those islands where *X. brasiliensis* was found, the results confirm this species as an exclusive *Rattus* parasite (Fig. 1). Canary Islands are the most palearctic region to this flea, unknown in North Africa.

Despite the fact that *X. guancha* has displayed the greatest global prevalence in this study, it has only been detected on Fuerteventura, Lanzarote and La Graciosa, eastern islands with the most arid climate of the entire archipelago, and it has also only been seen to parasitize *M. musculus*. Beaucournu *et al.* (1989) describe this species from the specimens coming from Lanzarote on *R. rattus*. Consequently, this is the first time that *X. guancha* has been detected on islands other than Lanzarote and, as far as is currently known, it is restricted to some of the eastern Canary Islands, where it is endemic as a parasite of rats and mice. *X. guancha* is the endemic *Xenopsylla* in Canary Islands and is issued from *X. ramesis* a gerbils species distributed from Marocco to Turkey; its occurrence in easternmost Canary Islands is obviously natural and significant.

 The results of this study show that no *Xenopsylla* species has been found on La Palma and La Gomera; while in past studies *X. brasiliensis* has been reported on specimens of *R. rattus* from these islands (Beaucournu *et al.*, 1989), it was not found on specimens of *R. rattus* trapped in the present study. The island of Lanzarote shows the most qualitative and quantitative *Xenopsylla* flea richness, being the only island where the three *Xenopsylla* species was present. As was stated previously, this is the first time that *X. cheopis* and *X. brasiliensis* have been reported on this eastern island. Despite the fact that the three *Xenopsylla* species detected in this study have a similar mean intensity of parasitation, their prevalence is quite different, with *X. guancha* being the most prevalent. The eastern islands prospected demonstrated greater qualitative and quantitative *Xenopsylla* flea richness than the western islands, even though the number of rodents trapped was higher in the western islands (560 *versus* 328). Consequently, it would seem that the eastern islands’ climate and proximity to the African continent influence the abundance of *Xenopsylla* and, in some cases, influence their endemicity as *X. guancha*.
